# Assessment of mutual aid older care needs for older adults with multimorbidity in urban China: a Kano model-based study

**DOI:** 10.3389/fpubh.2025.1612187

**Published:** 2025-09-23

**Authors:** Zhe Zhang, Yongqian Chen, Marcos C. Ochoa

**Affiliations:** ^1^School of Nursing, Bengbu Medical University, Bengbu, Anhui Province, China; ^2^Department of Human Resources, The Third Affiliated Hospital of Anhui Medical University, Hefei First People's Hospital, Hefei, Anhui Province, China; ^3^The School of Nursing, The Philippine Women's University Manila, Manila, Philippines

**Keywords:** mutual aid, older care needs, multimorbidity, Kano model, population ageing

## Abstract

**Background:**

China faces escalating challenges in older care because of rapid population ageing and the high prevalence of multimorbidity among urban older adults. Traditional family-based and institutional care models are increasingly inadequate, necessitating innovative solutions like mutual aid older care. However, research gaps persist in understanding the prioritised needs of urban older with multimorbidity within mutual aid older care frameworks. This study evaluates and prioritises mutual aid older care needs for older adults with multimorbidity in urban Chinese communities, by integrating Maslow’s Hierarchy of Needs with the Kano Model.

**Methods:**

A cross-sectional survey was conducted among 240 older adults (aged ≥65, with ≥2 chronic conditions) across 25 urban communities in Hefei, China. Using a validated questionnaire, 43 service items across safety/health, daily life, learning/socialisation, spiritual, and entertainment domains were assessed. Kano Model categorisation and better-worse coefficient analysis were applied to classify needs (must-be, one-dimensional, attractive, indifferent) and rank priorities via sensitivity calculations.

**Results:**

Key must-be needs included cooking, washing hair, and psychological counselling, while one-dimensional priorities emphasised exercise and travelling. Attractive needs featured telemedicine and cultural activities (e.g., Red Song Club). Indifferent items, such as pest control, had minimal impact on satisfaction. The analysis highlighted the centrality of physical health services, mental health support, and social engagement in enhancing quality of life.

**Discussion:**

Urban older adults with multimorbidity prioritise integrated care models addressing both basic survival needs and higher-order psychosocial well-being. Mutual aid older care frameworks must therefore balance healthcare accessibility with opportunities for social participation and emotional fulfilment. Future policies should adopt evidence-based, culturally tailored strategies to optimise resource allocation and foster sustainable ageing-in-place solutions. Limitations include geographic specificity and cross-sectional design, warranting broader longitudinal and mixed-method research.

## 1 Introduction

Population ageing has emerged as a global challenge, profoundly impacting socio-economic systems, healthcare infrastructure, and intergenerational dynamics (1). China, with over 209 million individuals aged 65 and above—accounting for 14.9% of its population—in 2022, faces accelerated demographic shifts, as it is expected to transition into a ‘moderately ageing society’ by 2030 (2). This demographic shift coincides with the increasing incidence of long-term health conditions and overlapping chronic illnesses (individuals living with multiple persistent medical issues), which particularly impact older populations (3). Studies indicate that 44.1% of older Chinese citizens experience multiple chronic conditions, worsening risks of physical limitations, diminished life quality, and earlier mortality (4, 5). Simultaneously, traditional family-based care systems face pressures owing to urban migration, smaller household sizes, and differing lifestyle expectations between generations, creating an urgent need for new solutions to older care gaps (6).

In response, mutual aid older care—arrangements that involve older adults assisting each other through shared resources and local community networks—has emerged as a viable alternative. This model emphasises active ageing, enabling older adults to shift from being passive care receivers to engaged contributors (7). International models—for instance, multigenerational households in Germany and community-based initiatives in the United States, demonstrate the potential to strengthen social connections and reduce reliance on formal institutions (8, 9). In China, trial programmes in cities and rural areas have revealed positive outcomes in addressing isolation, enhancing psychological well-being, and easing family care responsibilities (10). However, existing research primarily examines rural settings or general groups of older adults; thus, urban communities, especially those dealing with multiple health conditions, have been hitherto overlooked (11, 12).

Current studies identify structural obstacles limiting the expansion of mutual aid models in urban China, including inadequate policy frameworks, financial resource limitations, supply–demand imbalance, and limited engagement of older adults owing to scepticism or insufficient awareness (13, 14). Furthermore, while research recognises the specific difficulties posed by multiple chronic conditions—such as complicated care requirements and greater resource needs—a notable gap remains in understanding how these challenges interact with mutual aid systems (15). Existing analyses often use qualitative methods or overlook variations between older adult subgroups, leading to oversimplified suggestions that lack practical depth (16).

Therefore, the present study aims to employ Maslow’s Hierarchy of Needs as the primary theoretical framework to comprehensively assess the mutual support needs of older adults with multiple chronic conditions through a multidimensional questionnaire survey. This investigation systematically examines five critical dimensions—safety and health requirements, life requirements, learning and social needs, spiritual needs, and entertainment needs—while conducting an internal prioritisation analysis to identify hierarchical demand patterns. The ultimate objective is to establish an evidence-based hierarchy that addresses the most urgent requirements for multimorbid older adults in mutual-aid older care scenarios, thereby providing empirical guidance for targeted service optimisation.

This study adopted Maslow’s Hierarchy of Needs as the theoretical foundation for qualitative data collection. The framework conceptualises human needs as a five-tiered hierarchical pyramid, progressing from physiological needs (base level) to safety needs, love/belonging, esteem needs, and self-actualisation (apex level). As a seminal psychological theory of motivation, it posits that human needs follow a sequential hierarchy, with survival-oriented physiological requirements forming the foundational layer, while self-actualisation—the pursuit of personal potential—occupying the highest tier (17). Building on this framework, the research investigates mutual aid needs among urban-dwelling older adults with multimorbidity. It not only emphasises the fulfilment of basic physiological necessities but also addresses higher-order needs, such as emotional support, cultural engagement, and recreational activities. By prioritising these needs based on urgency, the study proposes stratified intervention strategies to address the most critical older care gaps.

**Figure 1:**
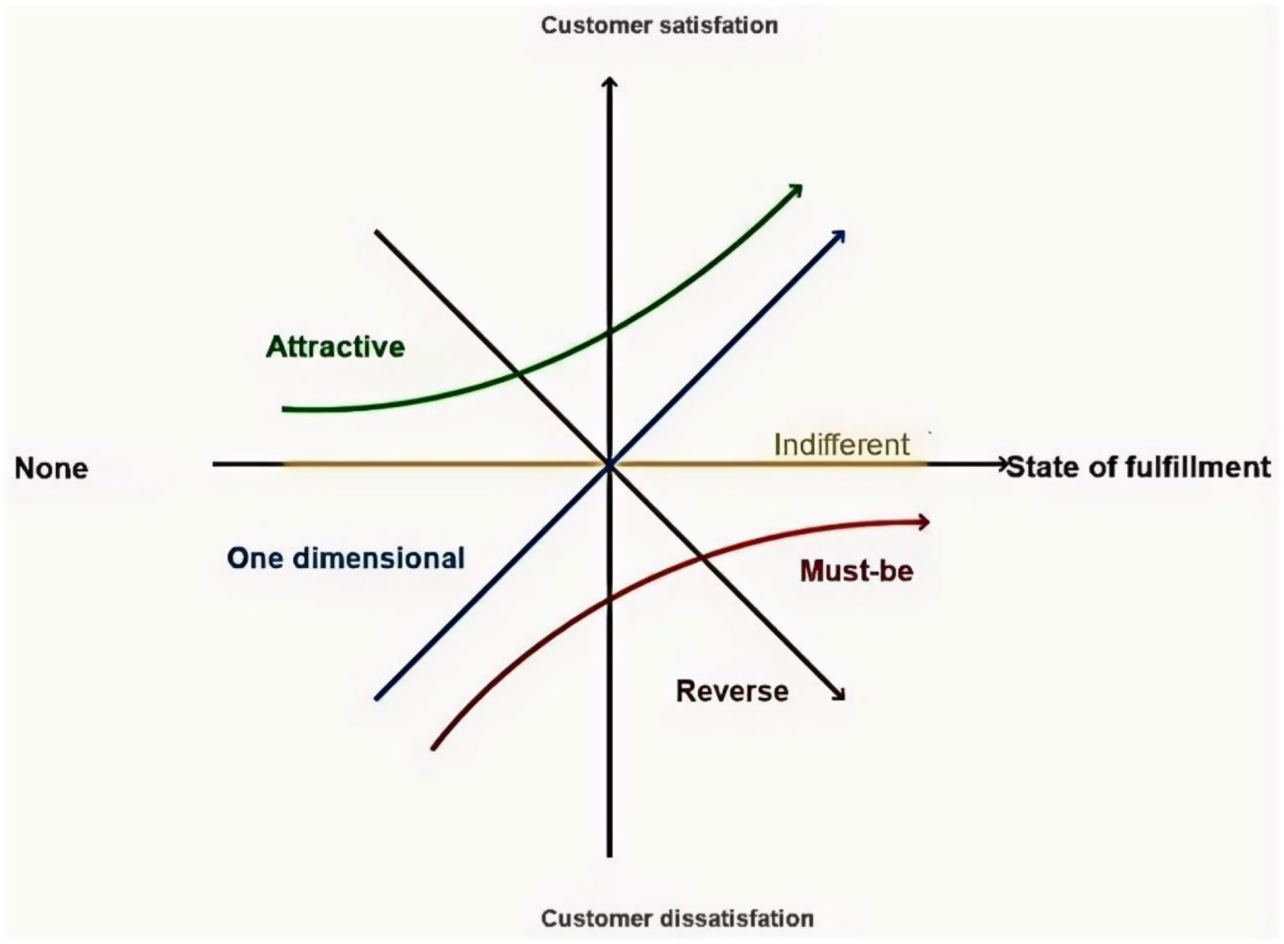
The Kano model.

The Kano Model is a method proposed by Noriaki Kano to identify quality, customer needs, and customer satisfaction attributes in quality management, industrial engineering, and business administration (Figure 1). Considered an effective tool for identifying and prioritising the individual needs of patients, it has been extended to medical quality management studies such as patient satisfaction, the need for remote care services for older adults in the community, and more (18). In this study, the Kano Model guides the prioritisation of strategies, ensuring that resource allocation aligns with the mutual aid older care needs of older adults with multimorbidity in urban China.

## 2 Materials and methods

### 2.1 Participants

The participants in this study were older adults living in 25 urban communities in Hefei, Anhui Province, China. The inclusion criteria of the study were: (1) voluntary participation after signing the informed consent form; (2) age ≥ 65 years; (3) at least 1 year of residence within the community; (4) multimorbidity with two or more chronic diseases; (5) mentally and intellectually normal, with the ability to understand and answer questions correctly; and (6) no limitation to physical mobility. The exclusion criteria were older adults with multimorbidity within the urban community who do not meet the above inclusion criteria. Such participants were automatically excluded from the study.

### 2.2 Sampling method and sample size

Purposive sampling was the method employed in the study. In multivariate analyses, the sample size should ideally be 5–10 times that of the study factor. A total of 43 influencing factors were included in the questionnaire used in this study. The estimated minimum sample size was 43 × 5 = 215 cases, while the estimated maximum sample size was 43 × 10 = 430 cases. We collected not less than 215 cases, considering time and conditions. A total of 244 participants were selected for the first phase of the survey study. Ultimately, 240 valid questionnaires were collected.

### 2.3 Research instruments

#### 2.3.1 Demographic questionnaire

A self-designed instrument was used administered to collect participants’ basic information (e.g., age, gender, health status). Reliability and validity tests were deemed unnecessary owing to the instrument’s descriptive nature.

#### 2.3.2 Mutual aid older care service demand questionnaire

Adapted from Zhou et al. (19) with written authorisation, the Mutual Aid Older Care Service Demand Questionnaire assessed five categories (safety/health, daily life, learning/socialisation, spiritual, and entertainment needs) aligned with Maslow’s Hierarchy of Needs. It comprised 43 paired items (positive and negative sub-questions), each rated on a 5-point Likert scale (ranging from “like” to “dislike”), as shown in Table 1. Responses were mapped to Kano attributes to classify service preferences. For example, paired questions—such as “How would you feel about having/ not having a home hair wash service?”—allowed the identification of critical, attractive, or indifferent needs.

**Table 1:** Data coding for Kano questionnaire responses.

Option	Like	Take it for granted	Indifferent	Reluctant	Dislike
Value	1	2	3	4	5

### 2.4 Reliability and validity analysis

The questionnaire’s reliability and validity were rigorously tested. Cronbach’s *α* coefficients were interpreted as follows: 0.6–0.7 (acceptable), 0.7–0.8 (good), and >0.8 (excellent). Values below 0.6 necessitated scale revision. An SPSS 25.0 analysis revealed an overall Cronbach’s *α* of 0.905, confirming high internal consistency. To determine validity, the Kaiser-Meyer-Olkin (KMO) measure (>0.9 = excellent; 0.8–0.9 = good; 0.7–0.8 = acceptable) and Bartlett’s sphericity test were applied. The questionnaire revealed a KMO value of 0.759 and a significant Bartlett’s test (χ^2^ = 6830.828, *p* < 0.001), indicating adequate validity for factor analysis at *p* < 0.05.

### 2.5 Analysis and interpretation

#### 2.5.1 Descriptive statistics

Demographic data were analysed using SPSS 26.0, with categorical variables presented as frequencies (percentages).

#### 2.5.2 Kano model classification

The evaluation of the Mutual Aid Elderly Care Needs Questionnaire was based on the results of the questionnaire for the classification of service needs. Statistical analyses were performed based on these results, and the attribute with the largest proportion was considered the requirement classification. A total of 25 permutations and combinations of each set of forward and negative problems were counted, as shown in Table 2.

**Table 2:** A Kano analysis evaluation form.

Functions/Services		Negative question				
		Dislike	Reluctant	Indifferent	Take it for granted	Like
Forward question	Dislike	Q	R	R	R	R
	Reluctant	M	I	I	I	R
	Indifferent	M	I	I	I	R
	Take it for granted	M	I	I	I	R
	Like	O	A	A	A	Q

A, Attractive attribute; O, One-dimensional attribute; M, Must-be attribute; I, Indifferent attribute; R, Reverse attribute; Q, Questionable results.

#### 2.5.3 Better-worse coefficient analysis

Satisfaction (Better/SI) and dissatisfaction (Worse/DSI) coefficients were calculated to rank service priorities, as follows:


Better/SI=(A+O)/(A+O+M+I)

$$\text{Worse}/\mathrm{DSI}=-1^{*}(O+M)/(A+O+M+I)$$

Higher better values indicated stronger satisfaction enhancement, while higher worse values reflected greater risk of dissatisfaction.

#### 2.5.4 Sequencing mutual aid older care service demands

The mutual older care demand prioritisation model, based on the element screening method, constructs an SI (better value) versus DSI (absolute worse value) matrix. Element (radius 0.707) and key element (radius 1.061) selection lines are plotted as quarter-arc boundaries from the origin. Demand priorities are classified by calculating the sensitivity (R-value)—the perpendicular distance from discrete points to the element selection line: right-side demands (points to the right of the key element line) require immediate improvement (highest priority); left-side demands can be deferred based on practical conditions. Higher sensitivity values indicate greater urgency, guiding resource allocation to address critical needs first. This spatial positioning system quantifies demand hierarchy, optimising resource distribution for older care services.

### 2.6 Ethical considerations

This study was approved by the Ethics Review Committee of the Philippine Women’s University. It adhered to the ethical standards pertaining to obtaining informed consent in writing and reading and checking the questionnaire before the participant completes it, especially if the participant chooses to remain anonymous. The considerations respected participants’ intention or reason for participating in the study, included an explanation of the study process, and affirmed the participants’ right to refuse to join the study and voluntarily withdraw from the study for any reason, at any stage, without penalty.

## 3 Results

### 3.1 General information

The demographic profile of older adults with multimorbidity in urban China reveals a male-dominated cohort (52.9%), predominantly aged between 65 and 80 years (88.75%), with limited educational attainment (55.8% primary education or below). This aligns with studies linking low health literacy to challenges in chronic disease management among less-educated seniors (20). Marital status reveals a potential familial support system (87.5% married), although widowed individuals (12.5%) highlight isolation risks, compounded by low social participation, with 40% never having engaged in social activities and 52.08% participating only occasionally. Such disconnection exacerbates mental and physical health decline, particularly in multimorbidity contexts (21).

Financial vulnerability is evident, with 54.58% relying on pensions and 42.08% on family support, reflecting systemic gaps in economic security amid rising healthcare costs (22). These challenges mirror broader trends in urban older care, with ageing populations facing intersecting burdens of health decline, social isolation, and financial strain (Table 3).

**Table 3:** Participants’ demographic profile and characteristics.

Factor	Catalogue	Frequency	Percentage
Sex (*n* = 240)	Male	127	52.9
	Female	113	47.1
Age (*n* = 240)	65–70 years old	95	39.58
	71–80 years old	118	49.17
	81–90 years old	27	11.25
Educational level (*n* = 240)	Primary school and below	134	55.8
	Junior high school	67	27.92
	Senior middle school/polytechnic school	28	11.67
	College/junior college and above	11	4.61
Marital status (*n* = 240)	Widow/widower	30	12.5
	Divorced	0	0
	Unmarried	0	0
	Married	210	87.5
Number of chronic diseases (*n* = 240)	2	213	88.75
	3	27	11.25
	4	0	0
	>4	0	0
Social participation (*n* = 240)	Active participation	19	7.92
	Occasional participation	125	52.08
	No participation	96	40.00
Financial resources (*n* = 240)	Pension	131	54.58
	Family funding	101	42.08
	Work income	8	3.34
	Others	0	0

### 3.2 Kano attributes and calculation of the better-worse coefficient

**Figure 2:**
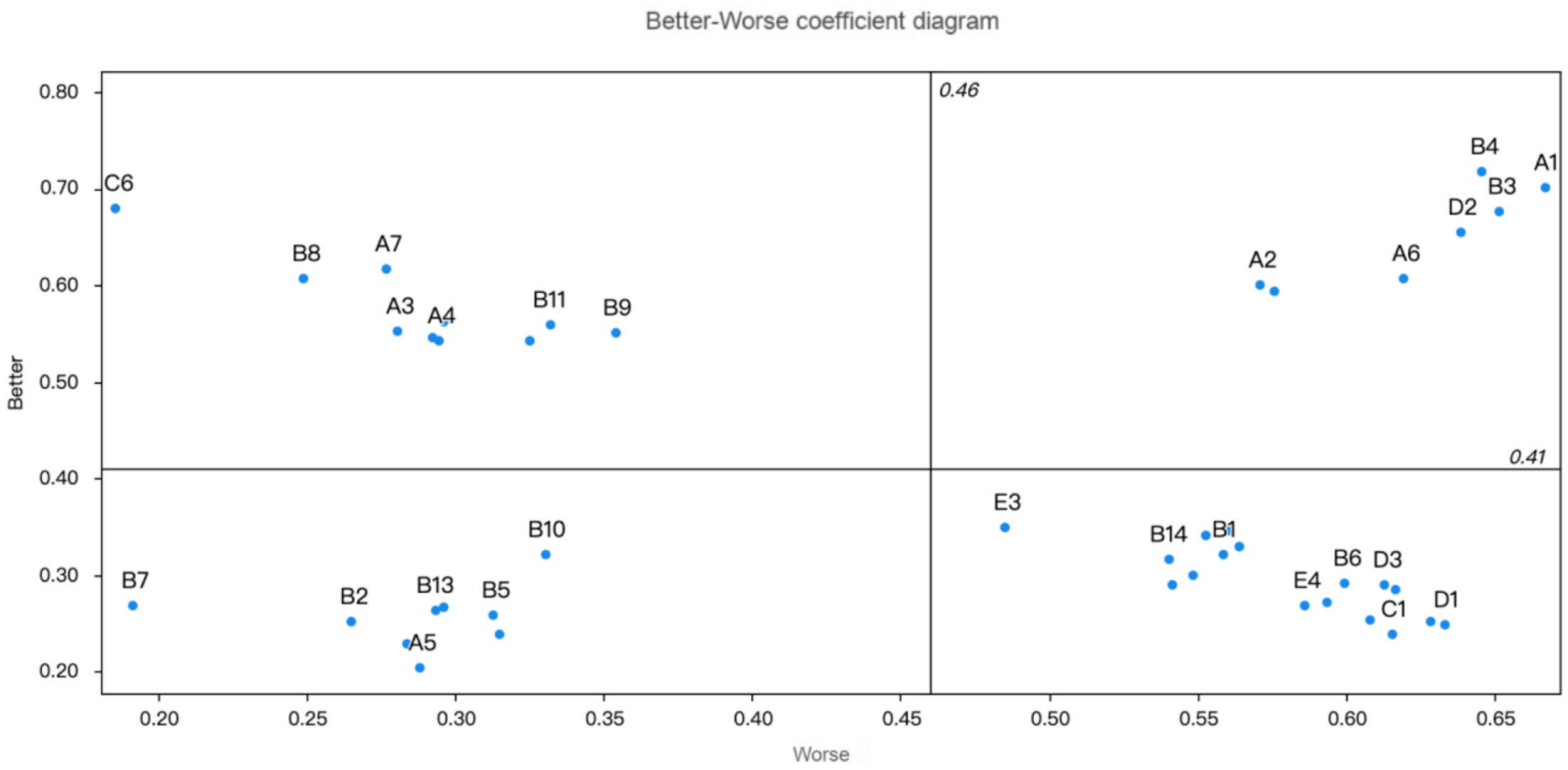
Better-worse coefficient diagram.

The priority order of mutual aid older care needs for older adults with multimorbidity in urban communities, as depicted in Table 4, categorises care needs into four groups: must-be (M), one-dimensional (O), attractive (A), and indifferent (I), emphasising the relative importance of various services in enhancing the quality of life for older adults, as determined by the better–worse coefficient analysis. Specifically, B1, B6, B14, C1, C2, C5, C4, C7, C9, D1, D3, D4, D5, E2, E3, E4, and E5 were identified as must-be qualities; A1, A2, A6, B3, B4, C6, and C8 as one-dimensional qualities; A3, A4, A7, B8, B9, B11, B12, B17, C6, and E1 as attractive qualities; and A5, B2, B5, B7, B10, B13, B15, B16, and C3 as indifferent qualities (Table 4; Figure 2).

**Table 4:** Kano attributes and better–worse coefficients of demand for older care older adults with multimorbidity.

Dimension	No.	Items	Number code	Categorical attribute	Better	Worse
Safety and health requirements	1	Exercise	A1	O	70.04%	−66.67%
	2	Rehabilitation training	A2	O	60.00%	−57.08%
	3	In-home medical service	A3	A	55.23%	−28.03%
	4	Telemedicine	A4	A	54.20%	−29.41%
	5	Taking medicine	A5	I	20.34%	−28.81%
	6	Boiling traditional Chinese medicine	A6	O	60.67%	−61.92%
	7	Caring for the sick	A7	A	61.70%	−27.66%
Life requirements	1	Cooking	B1	M	32.08%	−55.83%
	2	Food distribution	B2	I	25.21%	−26.50%
	3	Hair cutting	B3	O	67.65%	−65.13%
	4	Travelling	B4	O	71.73%	−64.56%
	5	Brushing teeth	B5	I	25.83%	−31.25%
	6	Washing hair	B6	M	29.11%	−59.92%
	7	Bathing	B7	I	26.81%	−19.15%
	8	Cleaning	B8	A	60.70%	−24.89%
	9	Repairing household appliances	B9	A	55.02%	−35.37%
	10	Pest controlling	B10	I	32.17%	−33.04%
	11	Finding items	B11	A	55.88%	−33.19%
	12	Pick up express	B12	A	54.27%	−32.48%
	13	Mowing the lawn	B13	I	26.29%	−29.31%
	14	Walking the dog	B14	M	31.65%	−54.01%
	15	Feeding birds	B15	I	22.88%	−28.39%
	16	Purchasing living goods	B16	I	23.83%	−31.49%
	17	Legal consulting	B17	A	54.66%	−29.24%
Learning and social needs	1	Tea party for older adults	C1	M	23.93%	−61.54%
	2	Cooking competition	C2	M	29.00%	−54.11%
	3	Foreign language learning	C3	I	26.67%	−29.58%
	4	Sightseeing	C4	M	34.18%	−55.27%
	5	Party dating	C5	M	25.43%	−60.78%
	6	Red Song Club	C6	A	67.93%	−18.57%
	7	Life skills learning	C7	M	29.96%	−54.85%
	8	Instructions for using electronic products	C8	O	59.31%	−57.58%
	9	Learning craft making	C9	M	34.62%	−55.98%
Spiritual needs	1	Chatting	D1	M	24.89%	−63.32%
	2	Psychological counselling	D2	O	65.53%	−63.83%
	3	Listening to music	D3	M	28.94%	−61.28%
	4	Watching movies	D4	M	27.12%	−59.32%
	5	Voluntary activities	D5	M	32.91%	−56.41%
Entertainment needs	1	Practicing calligraphy	E1	A	56.25%	−29.58%
	2	Square dancing	E2	M	28.45%	−61.64%
	3	Going to the park	E3	M	34.93%	−48.47%
	4	Practicing Tai chi	E4	M	26.78%	−58.58%
	5	Learning musical instruments	E5	M	25.21%	−62.82%

A, Attractive attribute; O, One-dimensional attribute; M, Must-be attribute; I, Indifferent attribute; R, Reverse attribute.

### 3.3 Internal priority order calculation results

Finally, we derived the internal priority of each function/service item by using the important sensitivity calculation:


$$R=\sqrt{{|\text{Better}|}^{2}+{|\text{Worse}|}^{2}}$$

**Figure 3:**
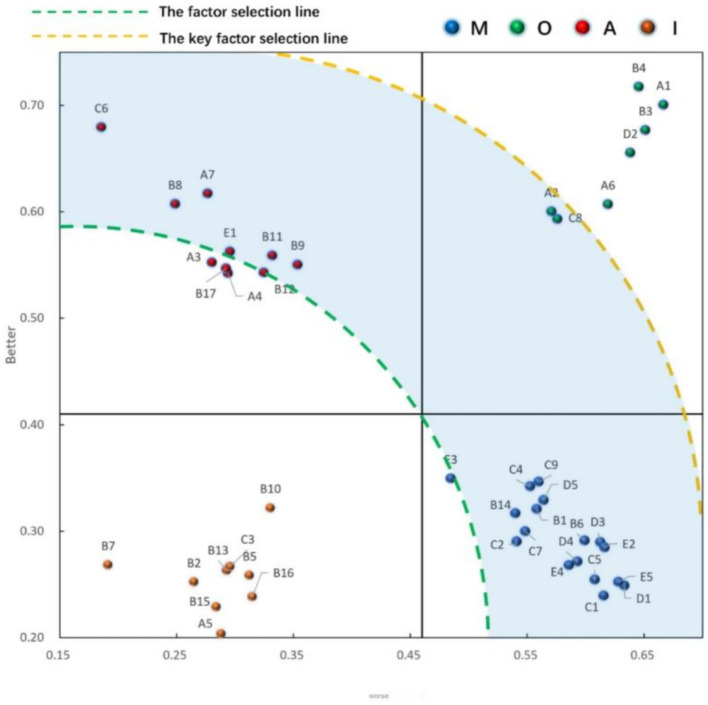
Sensitivity comparison matrix of improved factor and key factor.

The internal priority calculation results for each function/service item are presented in Table 5 and Figure 3. The top five improvement elements are exercise, boiling traditional Chinese medicine, travelling, haircutting, and psychological counselling. These findings suggest that community managers should concentrate on these five needs of older adults with multiple chronic conditions and prioritise enhancements based on the community department’s actual situation.

**Table 5:** The internal priority order calculation results of demand for older care among older adults with multimorbidity.

Dimension	No.	Items	Number code	Categorical attribute	R	Order
Safety and health requirements	1	Exercise	A1	O	96.70%	1
	2	Rehabilitation training	A2	O	82.80%	6
	3	In-home medical service	A3	A	61.90%	31
	4	Telemedicine	A4	A	61.70%	32
	5	Taking medicine	A5	I	35.30%	42
	6	Boiling traditional Chinese medicine	A6	O	86.70%	5
	7	Caring for the sick	A7	A	67.60%	13
Life requirements	1	Cooking	B1	M	64.40%	25
	2	Food distribution	B2	I	36.60%	40
	3	Hair cutting	B3	O	93.90%	3
	4	Travelling	B4	O	96.50%	2
	5	Brushing teeth	B5	I	40.50%	36
	6	Washing hair	B6	M	66.60%	14
	7	Bathing	B7	I	32.90%	43
	8	Cleaning	B8	A	65.60%	18
	9	Repairing household appliances	B9	A	65.40%	19
	10	Pest controlling	B10	I	46.10%	35
	11	Finding items	B11	A	65.00%	22
	12	Pick up express	B12	A	63.20%	27
	13	Mowing the lawn	B13	I	39.40%	39
	14	Walking the dog	B14	M	62.60%	28
	15	Feeding birds	B15	I	36.50%	41
	16	Purchasing living goods	B16	I	39.50%	38
	17	Legal consulting	B17	A	62.00%	30
Learning and social needs	1	Tea party for older adults	C1	M	66.00%	15
	2	Cooking competition	C2	M	61.40%	33
	3	Foreign language learning	C3	I	39.80%	37
	4	Sightseeing	C4	M	65.00%	23
	5	Party dating	C5	M	65.90%	16
	6	Red Song Club	C6	A	70.40%	8
	7	Life skills learning	C7	M	62.50%	29
	8	Instructions for using electronic products	C8	O	82.70%	7
	9	Learning craft making	C9	M	65.80%	17
Spiritual needs	1	Chatting	D1	M	68.00%	9
	2	Psychological counselling	D2	O	91.50%	4
	3	Listening to music	D3	M	67.80%	11
	4	Watching movies	D4	M	65.20%	21
	5	Voluntary activities	D5	M	65.30%	20
Entertainment needs	1	Practicing calligraphy	E1	A	63.60%	26
	2	Square dancing	E2	M	67.90%	10
	3	Going to the park	E3	M	59.70%	34
	4	Practicing Tai chi	E4	M	64.40%	24
	5	Learning musical instruments	E5	M	67.70%	12

A, Attractive attribute; O, One-dimensional attribute; M, Must-be attribute; I, Indifferent attribute; R, Reverse attribute.

## 4 Discussion

The findings of this study provide critical insights into the prioritised needs of older adults with multimorbidity in urban Chinese communities under the mutual aid older care framework. Our investigation identified 17 must-be demands, 7 one-dimensional demands, 10 attractive demands, and 9 indifferent demands. We also identified five factors that urgently require improvement while sorting internal priorities. By integrating the Kano Model with Maslow’s Hierarchy of Needs, this analysis highlights the multidimensional nature of older care requirements, emphasising the interplay between basic survival needs and higher-order psychosocial well-being.

### 4.1 Safety and health requirements

Exercise and rehabilitation training emerged as one-dimensional (O) attributes, indicating their direct correlation with satisfaction. This aligns with existing research, which emphasises the importance of physical activity in maintaining older adults’ health and mobility, particularly for those with multiple chronic conditions. Exercise programmes help manage conditions such as hypertension, arthritis, and diabetes, all of which are common among older adult populations (23, 24). Notably, in-home medical services and telemedicine were categorised as attractive (A) attributes, reflecting their growing role in urban older care. Research reveals that telemedicine has become an increasingly important tool in managing chronic diseases remotely, especially in urban settings where healthcare infrastructure can support such technology (25). However, medication management, classified as indifferent (I), suggests that older individuals perceive it as routine rather than as a service requiring external support, underscoring the need for targeted health literacy programmes.

### 4.2 Life requirements

Must-be (M) attributes, such as cooking and hair washing, underscore the foundational role of daily living assistance. Research reveals that older adults, particularly those with multimorbidity, heavily rely on such basic care activities to maintain their quality of life, as these tasks become harder to manage independently with advancing age and chronic illness (26). Conversely, attractive attributes such as cleaning and repairing appliances underscore the value of convenience-enhancing services, which align with the provision of a clean and functional home environment. This is crucial to maintain the comfort and safety of older adults, contributing to their overall sense of security and well-being (27). The indifferent response to pest control and food distribution suggests that such tasks may already be managed informally, emphasising the need for community needs assessments to avoid resource misallocation.

### 4.3 Learning and social needs

Social activities such as tea parties for older adults and cooking competitions were classified as must-be attributes, reinforcing the critical role of social engagement in mitigating isolation. Although their presence does not significantly elevate satisfaction, their absence leads to considerable dissatisfaction, suggesting that these activities are crucial to maintain social engagement and prevent isolation among older adults (28). The Red Song Club, an attractive attribute. Highlights the cultural significance of activities that involve engaging in nostalgic and collective experiences, which provides a strong sense of community and emotional satisfaction. Such social activities help older adults relive shared histories and foster social bonds, contributing to emotional well-being and social connection (29, 30). Meanwhile, the indifferent response to foreign language learning suggests a preference for practical, health-focused interventions over skill acquisition. This aligns with broader findings that older adults in urban communities with multimorbidity prioritise social and recreational activities that are more immediately relevant to their cultural and social lives, rather than learning new skills they do not deem essential (20).

### 4.4 Spiritual needs

Psychological counselling, categorised as one-dimensional, highlights the unmet demand for mental health support in ageing populations. This aligns with existing research that highlights psychological support needs, particularly for older adults dealing with multiple chronic health issues, to help them manage stress, depression, and anxiety (31, 32). Listening to music and watching movies are important must-have attributes. The positive effect of music can lower anxiety levels and help with mental abilities, while watching movies offers stimulation and enjoyment, according to recent findings (33, 34). Volunteer work and casual conversations, both of which are essential attributes, reveal the necessity of ongoing social contact to fight loneliness and preserve purpose. In other words, volunteering helps older adults stay active while contributing to their communities, which research indicates boosts self-esteem and decreases feelings of isolation (35). Meanwhile, chatting is an easy but vital way to connect socially, addressing loneliness concerns common among those managing multiple health problems (36).

### 4.5 Entertainment needs

Among entertainment-related needs, calligraphy practice emerges as an appealing attribute owing to its creative expression and mental involvement. Studies suggest that activities like this can reduce anxiety levels, promote relaxation states, and support cognitive functions among older adults (37). As an art form with deep cultural roots, calligraphy strengthens cultural connections and feelings of personal accomplishment. Square dancing, classified as a fundamental attribute, remains widely popular among urban Chinese older adults, providing health benefits and social interaction chances. Moreover, regular practice is key for sustaining both body fitness and emotional wellness, as evidenced in recent analyses (38). Similarly, Tai chi exercises and musical instrument learning reveal positive impacts on well-being, although their effects differ across individuals. These observations underline how combining physical activities with mental engagement approaches can enhance the quality of life for seniors facing complex health challenges (24, 25).

Internal priority assessments identified five key need areas as urgent priorities for mutual aid older care among older adults with multiple chronic conditions: exercise, boiling traditional Chinese medicine, psychological counselling, travelling, and haircutting. The strong focus on exercise and boiling traditional Chinese medicine, both of which relate to basic health and safety requirements, reveals the need for expert guidance in physical recovery while simultaneously highlighting the lasting role of traditional medicinal practices in caring for older adults. The ongoing challenges of financial pressures combined with the emotional strain caused by long-term health issues underline the importance of supportive psychological services. Travelling, which serves as bridges between people, demonstrates potential benefits in reducing loneliness and stress through community interactions. The mention of haircutting services, which help maintain personal dignity, further illustrates how basic care needs intersect with emotional well-being.

The findings align with Maslow’s Hierarchy of Needs, showing that older adults prioritise survival needs while still valuing social belonging and personal growth opportunities. This dual focus challenges conventional care approaches that concentrate only on medical treatment for older adults, suggesting combined strategies that address whole-person wellness instead. Policy makers should consider practical solutions while developing community-based social initiatives to strengthen sustainable mutual aid older care models.

This research has several noteworthy limitations. First, the study design based on single-timepoint data collection limits cause–effect conclusions. Second, the geographical limitation to urban centres affects the generalisability of these findings to rural areas or regions with different cultural backgrounds. Third, reliance on self-reported information may lead to response inaccuracies, particularly among participants with memory challenges. Future studies should use long-term observation methods combined with multiple data types to not only track changing needs over time but also examine urban–rural differences. Moreover, exploring technology-based solutions could help address scalability issues in community-led older care systems.

## 5 Conclusion

This research seeks to identify the care requirements for older adults dealing with multiple chronic conditions in urban Chinese communities through the integration of Maslow’s Hierarchy of Needs and the Kano Model. The findings from this investigation highlight a hierarchical structure in need prioritisation, such as exercise programmes and rehabilitation training, psychological support areas including counselling sessions and opportunities for social interaction, as well as culturally meaningful activities such as participation in Red Song Club groups or Tai chi practice. These elements collectively contribute to improving life quality among older adults with multimorbidity and provide precise guidance for the formulation of policies linked to mutual aid older care needs.

## Data Availability

The original contributions presented in the study are included in the article/supplementary material, further inquiries can be directed to the corresponding author.
